# Pneumoscrotum with extensive subcutaneous emphysema in traumatic perineal injury: A case report

**DOI:** 10.1002/ccr3.7932

**Published:** 2023-11-25

**Authors:** Tariq Siddiqui, Ruben Peralta, Muhamed Ibnas, Ayman El‐Menyar, Hassan Al‐Thani, Sandro Rizoli

**Affiliations:** ^1^ Department of Surgery, Trauma Surgery Hamad Medical Corporation Doha Qatar; ^2^ Department of Surgery School of Medicine Universidad Nacional Pedro Henríquez Ureña Santo Domingo Santo Domingo Dominican Republic; ^3^ Department of Surgery, Trauma and Vascular Surgery, Clinical Research Hamad Medical Corporation Doha Qatar; ^4^ Department of Clinical Medicine Weill Cornell Medical College Ar‐Rayyan Qatar

**Keywords:** blunt, case report, perineal injury, pneumoscrotum, subcutaneous emphysema, trauma

## Abstract

**Key Clinical Message:**

We present a rare case of pneumoscrotum with subsequent subcutaneous emphysema in penetrating perineal injury with a tangential wound. It is important to diagnose the underlying disease and treat the cause. An examination under anesthesia is crucial for the diagnosis and management of the set of injuries.

**Abstract:**

Pneumoscrotum with subcutaneous emphysema in traumatic perineal injuries is an alarming sign and may indicate life‐threatening intraabdominal injuries or necrotizing fasciitis. We reported a case of pneumoscrotum and extensive subcutaneous emphysema of the abdomen and chest 2 days after admission. Pneumoscrotum was not seen on the initial Computerized tomographic scan.

## Graphical abstract

Pneumoscrotum with subcutaneous abdominal and chest emphysema after perineal trauma.

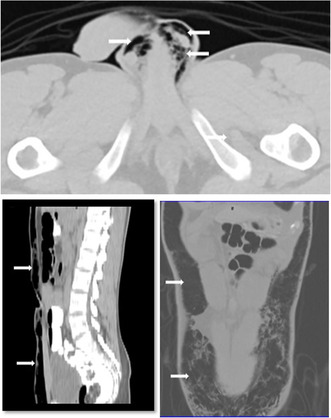

## 1 INTRODUCTION

Air in scrotum is a rare condition known as pneumoscrotum.^1^ Dagur et al. reported a rare case of extensive subcutaneous emphysema with pneumoscrotum in perineal injury.^2^ The source could be air containing cavities like sinuses, trachea, lungs, hollow viscus organs, or environmental air entering via a wound.^3^ Clinically, patient complaints could vary from mild pain and swelling of the affected area to extensive subcutaneous emphysema resulting in respiratory and cardiovascular compromise.^4^ The possible causes of subcutaneous emphysema include iatrogenic procedures, endoscopy, biopsy, chest drain, post‐surgery, trauma to chest, air contained organs, infectious disease, Fournier's gangrene, enterocutaneous fistula.^3^, ^4^ Penetrating perineal injury is a serious mechanism of injury and can be trivial or life‐threatening with the involvement of intra‐abdominal and pelvic organ injury.^5^

Patients with penetrating perineal injury need to be properly investigated with Computerized tomographic scan with intravenous contrast and oral or rectal contrast and then properly treated as per cause and on individual basis.^2^ This is a rare case with penetrating perineal injury diagnosed and treated at a level 1 trauma center.

## 2 CASE REPORT

A 33‐year‐old bicyclist male, with no significant past medical history, who was hit by a car and brought to the trauma center directly by the Emergency Medical Services with backache, left chest pain, and perianal pain. Primary survey was unremarkable showing stable vitals with no fever. Secondary survey revealed a Glasgow Coma Scale (GCS) of 15, good bilateral air entry, no external bruises or swelling. Abdomen was soft and non‐tender. Bleeding from a lacerated wound in the perineum (5 × 3 cm) between the scrotum and the anal verge was noted and controlled with a compression dressing. The anal tone was normal. Proctoscopy revealed no mucosal damage or bleeding. The blood investigation for the trauma panel was normal. Pan computerized tomography (CT) scan of the head, neck, abdomen, and chest was unremarkable. Basic trauma panel laboratory tests were normal. Initial CT scans of the abdomen and pelvis were unremarkable. The patient was admitted to the surgical ward for pain control and perineal wound management. On admission, he was started on empirical antibiotic therapy (amoxicillin and metronidazole).

The patient did not have any respiratory or urinary tract symptoms, and urinary analysis was not requested. The patient's wound was washed with normal saline and packed with gauze wet to dry dressing. The patient was seen on the next day and the plan was to continue dressing with sitz bath. Two days after admission, local examination showed emphysema of the penis and the scrotal area extending to the abdomen and lower chest with a hissing sound.

The wound looked healthy, with no blebs or active bleeding. The patient was afebrile with no change in vital signs. The white blood cell range was normal. The patient was not complaining of any abdominal pain other than subcutaneous emphysema. On follow‐up, his abdomen was soft, lax, non‐tender, and chest examination was normal.

**FIGURE 1:**
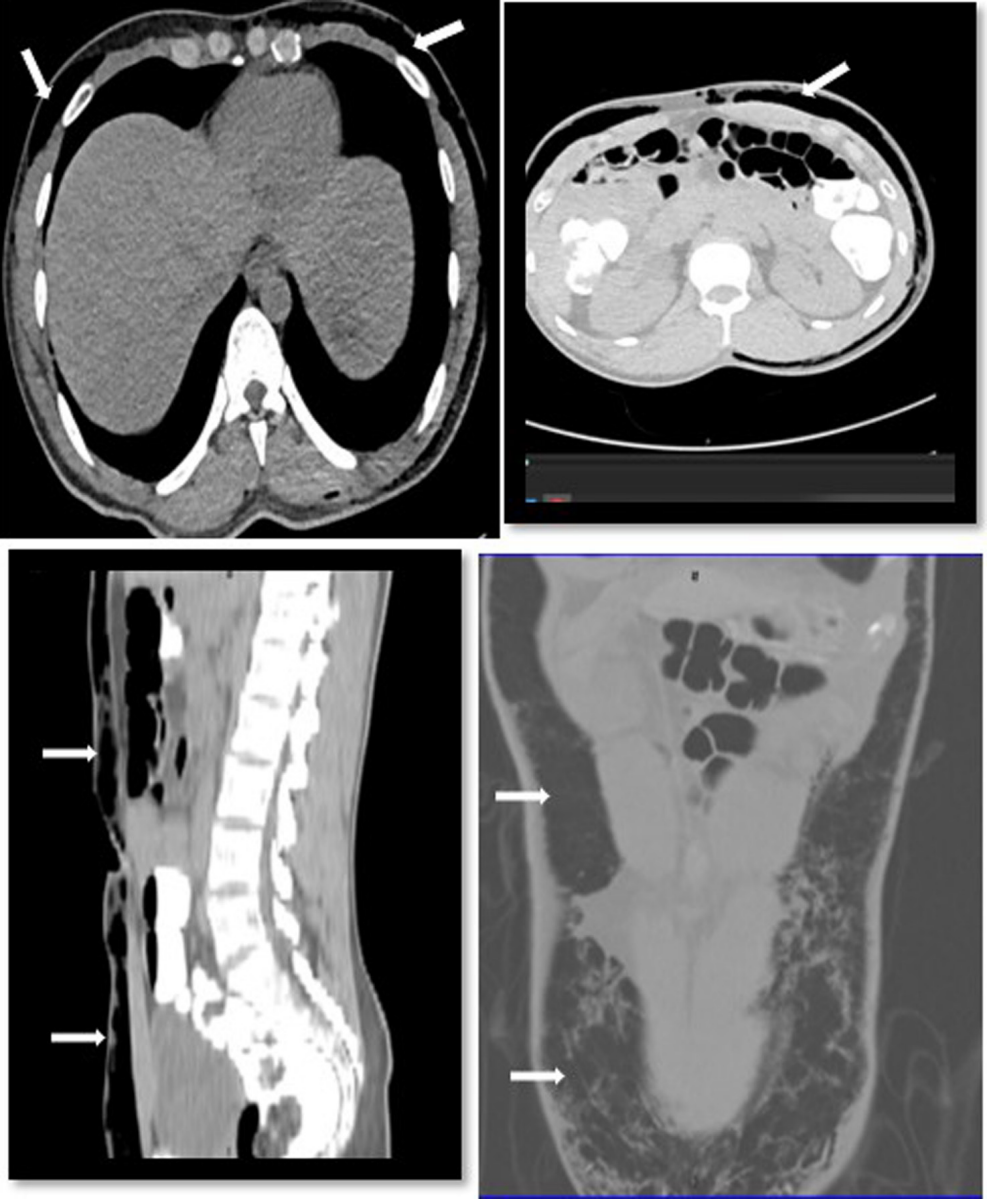
CT scan of chest, abdomen and pelvis showing subcutaneous emphysema.

**FIGURE 2:**
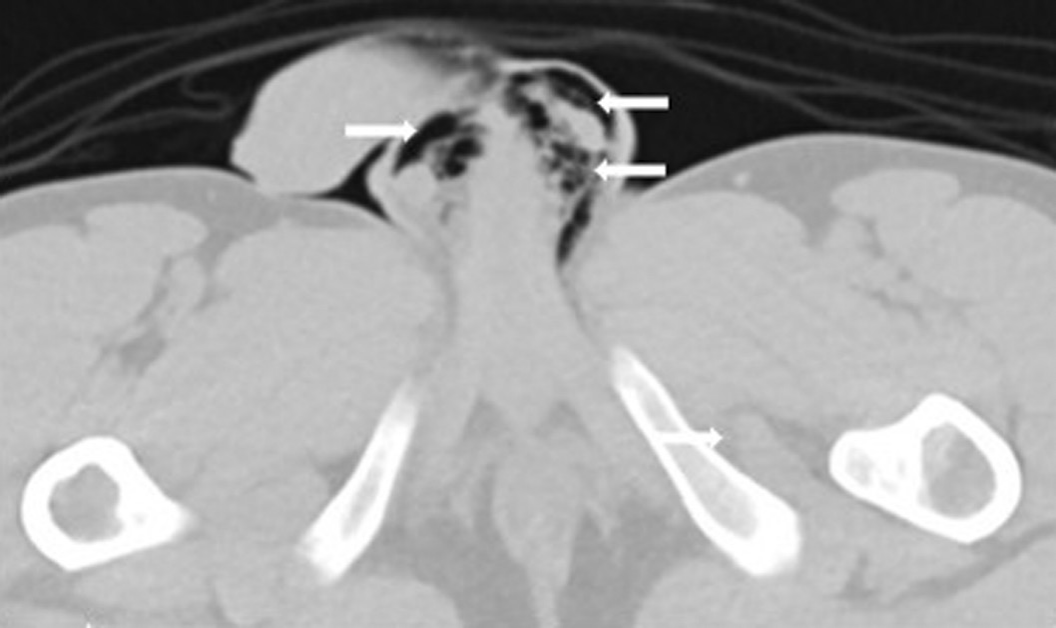
CT scan pelvis showing Pneumoscrotum.

Repeat CT abdomen and pelvis with rectal and IV contrast were performed. Rectal contrast showed opacification of the large bowel with no obvious extraluminal contrast leak. CT abdomen revealed neither intraperitoneal air nor free fluid. Subcutaneous emphysema was seen in the penoscrotal area extending to the abdomen till the lower chest (Figures 1 and 2). No pneumothorax, pneumomediastinum, or pneumoperitoneum was noted.

**FIGURE 3:**
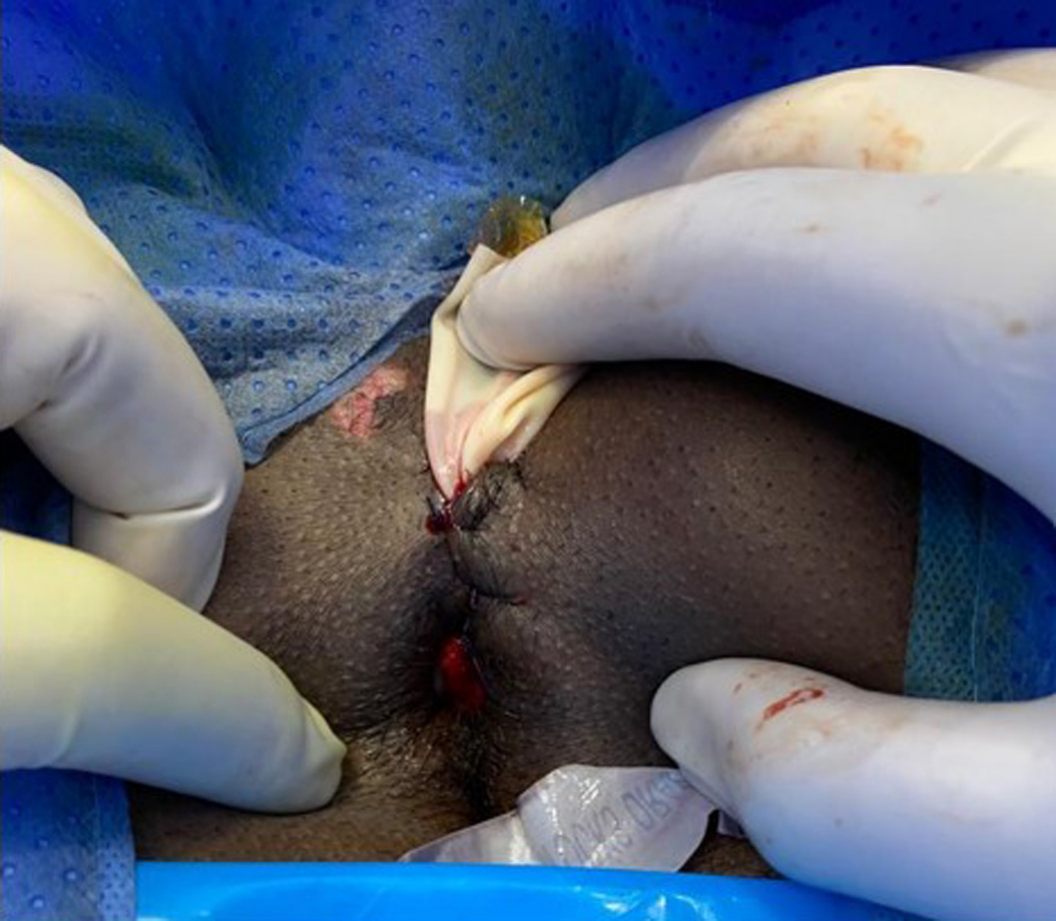
Perineal wound after closure with drain.

The patient was taken to the operating theater for wound exploration (Figure 3). Tangential wound in the lithotomy position at 1 o'clock position was seen with the last 3 cm of the wound extending under the skin piercing the dartos muscle (tunica dartos). Proctoscopy and sigmoidoscopy did not reveal any evidence of lower bowel injury of mucosa. Healthy fascia adherent with muscle, bleeding healthy tissues, and absence of any foul‐smelling pus ruled out necrotizing fascia.

The wound was washed with hydrogen peroxide and closed with Vicryl 3/0 with a penrose soft drain to rule out blood collection or pus. Subcutaneous emphysema of abdomen and lower chest wall resolved by dissolving into the subcutaneous tissue. The drain was removed on the 3rd post‐operative day. The patient was discharged home on the 6th day post‐admission. Patient was seen in the outpatient clinic 8 days after discharge. Pneumoscrotum and subcutaneous emphysema resolved completely, and the perineal wound healed well. He returned to normal activity with no reported related problems during the follow‐up visits up to 1 year.

## 3 DISCUSSION

Most of the reported cases of pneumoscrotum in literature are due to direct trauma to the scrotum. Our case is unique due to its presentation as a perineal injury. The wound, however, was tangential, extending to the underlying dratos muscle where the air made its way to the scrotum. Therefore, we suggest proper examination of perineal wounds under general anesthesia. We present a rare case of pneumoscrotum with subsequent subcutaneous emphysema of the abdomen and chest that was missed on initial clinical evaluation after perineal injury. A prior case of an elderly patient with chronic non‐healing scrotal wound presented with extensive emphysema and pneumoscrotum with bilateral pneumothorax which resolved after chest tube insertion, but the source of pneumothorax was not ascertained.^6^ In our case, the patient did not have a pneumothorax and the source of air was from the far‐away wound.

Laceration of the scrotum with massive scrotal subcutaneous and retroperitoneal emphysema is reported in the literature with a wound size of 1.5 × 1.5 cm with a metallic foreign body. The wound was managed conservatively and not closed. The patient returned 5 days later with a massive bilateral scrotal and suprapubic subcutaneous emphysema.^7^ Our case did not show any retroperitoneal air on the CT scan. Akil et al reported an extensive subcutaneous emphysema secondary to perineal ulcer (3 × 3cm) and a pneumoscrotum that was treated conservatively.^8^

There are two ways for air to reach the scrotum. The first one, is direct air or gas introduced into the scrotum from outside. The second way is air inside the thoracic cavity travels along the layer of Scarpa and campers. These two‐facia merge to form colles and dartos at the base of penis and scrotum, respectively. The air can travel from abdominal cavity to the abdominal wall by diffusion, then along the facial plane and into the scrotum. Subcutaneous emphysema and pneumoscrotum can easily be picked by x‐ray in most cases. CT scan can detect underlying cause. Ultrasonography is helpful in detecting inflammation of epididymis and testis. Pneumoscrotum by itself is self‐resolving but the underlying cause need to be diagnosed early and treated as it may lead to tension pneumothorax, bowel perforations or necrotizing fasciitis. Other causes are iatrogenic intervention.^4^ Furthermore, diagnostic, and therapeutic colonoscopy or endoscopy has been associated with pneumoscrotum suggestive of bowel perforation.^9^

Mukendi presented a case of blunt trauma in a patient who was intubated, and chest tube was placed for suspected pneumothorax. On arrival to hospital, pneumoscrotum was noted which improved after replacing the chest tube.^10^ El Ellani et al presented a case of pneumoscrotum with pelvic fracture, scrotal and perineal laceration that was managed by fixation of fractures and Delbet drain.^11^

The management includes treatment of the underlying cause, extensive surgical emphysema may need an infraclavicular drain.^9^ While chest tubes and laparotomy and debridement of wound and antibiotic is as per the pathology. Table 1 summarizes the reported cases of traumatic pneumoscrotum diagnosed with CT scan.^10^, ^11^, ^12^, ^13^, ^14^, ^15^, ^16^, ^17^, ^18^, ^19^, ^20^, ^21^, ^22^

**TABLE 1:** literature review of concurrent traumatic pneumoscrotum diagnosed with CT scan.

Reference	Age in years	Mechanism of injury	Cause	Other site of subcutaneous emphysema	Treatment
Lostoridis et al.^13^	82	Fall from stairs	Blunt chest trauma, pneumothorax and pneumoscrotum	Eyelid to abdominal wall	Chest tube, antibiotics
Su et al.^14^	44	Blunt chest trauma	Tracheal injury, pneumothorax and pneumoscrotum	Chest, abdomen, both legs	Repair of tracheal injury
Simaioforidis et al.^15^	22	Motorcycle accident	Pneumoscrotum and pneumothorax after blunt chest trauma	Chest and abdomen	Chest tube
Ural et al.^16^		Self‐inflicted	Injection of air in the penis	Chest and abdomen	Antibiotics
Mukendi et al.^10^	52	Blunt trauma to chest	Pneumoscrotum, pneumothorax	Chest and abdomen	Intercostal drain
Linda et al.^11^	30	Blunt heavy object	Pelvic fracture with perineal wound, pneumoscrotum	Face, neck, chest, abdomen, thigh	Fracture stabilization, external fixation, wound dressing, Delbet drains
Ravisagar et al.^18^	62	Road accident	Blunt chest trauma, pneumoscrotum	Chest and abdomen	Chest tube
Alvarez et al.^19^	57	Road accident	Blunt chest trauma, pneumoscrotum	Extensive emphysema from ocular zone to root of thigh	Chest tube
Peker et al.^20^	57	Motorcycle accident	Pneumothorax, pneumoscrotum	Chest	Chest tube
Lin et al.^21^	72	Fall	Pneumothorax, pneumoscrotum	Face to scrotum	Chest tube
Kono et al.^22^	32	Blunt heavy object	Pneumothorax, pneumoscrotum	Lower abdomen	Chest tube

## 4 CONCLUSION

Perineal trauma without pelvic fracture and anorectal injury may present with pneumoscrotum and extensive subcutaneous emphysema that could be benign condition. However, ruling out other serious causes by clinical examination and radiological investigation is needed to avoid unnecessary aggressive therapy. All perineal wounds should be examined under general anesthesia and sutured to avoid potential complications.

## AUTHOR CONTRIBUTIONS

**Tariq Siddiqui:** Conceptualization; data curation; writing – original draft. **Muhamed Ibnas:** Data curation; methodology. **Ruben Peralta:** Data curation; investigation. **Ayman El‐Menyar:** Supervision; writing – review and editing. **Hassan Al‐Thani:** Supervision; writing – review and editing. **Sandro Rizoli:** Conceptualization; writing – review and editing.

## FUNDING INFORMATION

None.

## CONFLICT OF INTEREST STATEMENT

The Authors declare that there is no conflict of interest.

## ETHICS STATEMENT

The Medical Research Center of Hamad Medical Corporation has granted permission (MRC‐04‐23‐208) for this case report to be published on condition that no patient identifiable data (including patient name and photograph) are included.

## CONSENT

A written consent was received from the patient.

## Data Availability

Data used to support the findings of this case are included within the article.
